# Prevalence of malnutrition and associated factors in children aged 6–59 months among rural dwellers of damot gale district, south Ethiopia: community based cross sectional study

**DOI:** 10.1186/s12939-017-0608-9

**Published:** 2017-06-26

**Authors:** Lamirot Abera, Tariku Dejene, Tariku Laelago

**Affiliations:** 1 Central statics agency, Addis Ababa, Ethiopia; 2 Department of population studies, Addis Ababa University, Addis Ababa, Ethiopia; 3 Department of health information technology, Hossana College of health science, Po. Box 159, Hossana, Ethiopia

**Keywords:** Malnutrition, Damot Gale, Rural dwellers, Children, Underweight, Wasting

## Abstract

**Background:**

Malnutrition remains one of the most common causes of morbidity and mortality among children throughout the world. This study aimed to assess prevalence of malnutrition and associated factors among children aged 6–59 months in Damot Gale, South Ethiopia.

**Methods:**

A community based cross sectional study was conducted on 398 children aged 6–59 months in the Damot Gale district. A two-stage cluster sample design was used to select kebele and households. Anthropometric measurements and structured questionnaires were used to collect data. Bivariate and multivariate logistic regression was done by using SPSS version 20.

**Results:**

The results of this study indicated that 27.6% of children were under-weight and 9% were wasted. Being male (AOR: 1.90; 95% CI: (1.10–3.32), children with shorter birth interval (AOR:2.89;95% CI: (1.23–6.80), children who had sickness some times for past 2 weeks (AOR:0.42; 95% CI:(0.10–0.93) and children whose mothers attended ANC (AOR:0.29; 95% CI: (0.16–0.52) were associated with underweight. Children whose mother’s main occupation was non-farm (AOR: 7.06;95% CI: (1.31–38.21), presence of diarrhea (AOR:39.5, 95% CI: (13.68–114.30), and children whose mothers attended ANC (AOR:0.18,95% CI: (0 .18 (0.07–0.45) were associated with wasting.

**Conclusion:**

The prevalence of malnutrition in the study area was high. Health extension workers and stakeholders should give due concern on promotion of proper nutrition in the community.

## Background

Malnutrition most often refers to under nutrition, resulting from inadequate consumption, poor absorption or excessive loss of nutrients, but the term can also encompass over-nutrition. Underweight, based on weight for-age, is a composite measure of stunting and wasting and is recommended as the indicator to assess changes in the magnitude of malnutrition over time. Wasting represents a recent failure to receive adequate nutrition and may be affected by recent episodes of diarrhea and other acute illnesses. Wasting indicates current or acute malnutrition resulting from failure to gain weight or actual weight loss [1].

Malnutrition remains one of the most common causes of morbidity and mortality among children throughout the world. It has been responsible, directly or indirectly, for 60% of the 10.9 million deaths annually among children under 5. Over two-thirds of these deaths, which are often associated with inappropriate feeding practices, occur during the first year of life [2, 3]. In Ethiopia, the prevalence of underweight and wasting is 29% and 10%, respectively. The prevalence of underweight is 16% in urban areas and 30% in rural areas. In SNNPR (south nations nationalities people region), 28% of children are underweight, and 7.6% are wasted [4].

The malnutrition status in a given population depends on many factors: the political and economic situation, the level of education and sanitation, the season and climate conditions, food production, cultural and religious condition, food customs, breast-feeding habits, prevalence of infectious diseases, the existence and effectiveness of nutrition programs and the availability and quality of health services [5].

Malnutrition during childhood is a result of a wide range of factors, most of which relate to unsatisfactory food intake or severe and repeated infections, or a combination of the two. The most frequently suggested causes of malnutrition are: poverty, low parental education, lack of sanitation, low food intake, diarrhoea and other infections, poor feeding practices, family size, short birth intervals, maternal time availability, child rearing practices and seasonality. There are also economic, social, and cultural causes of malnutrition, which underscore the close link between malnutrition [6].

A study done in Bule Hora district of Oromia region and in pastoral community of Dollo Ado district, Somali region, showed males children were more likely to be underweight than females [7, 8]. Children with lower preceding birth interval are at higher risk of underweight [9, 10]. Studies done in developing countries showed receipt of ANC (antenatal care) was negatively associated with both underweight and wasting [9, 11, 12]. A study conducted in south region of Ethiopia, showed children who had no sickness for the past 2 weeks from the study period were found to have decreased risk of underweight than children who were always sick [13].

The likelihood of underweight for children is higher when breast feeding is initiated more than an hour after birth as compared to children who were put to breast immediately after birth [14]. The study done in Bule Hora district of Oromia and Gondar, showed the presence of diarrhea was associated with wasting [7, 15].

The causes and determinants of children malnutrition are complex, interrelated and multidimensional. Therefore, if the present prevalence of malnutrition in children is to be reduced, then it is important that the most important causes of malnutrition should be understood. Besides this, assessing the nutritional status of children is an essential part of monitoring their health status and providing data for accurate planning and implementation of interventions to reduce morbidity and mortality associated with malnutrition. Thus, this paper focused on examining the prevalence and determinant factors of child malnutrition among children aged 6–59 months in rural parts of Damot Gale district. This study also provided evidence of a link between care during pregnancy and childhood malnutrition in the district. This is important for planning because, it suggests another benefit of caring for women during pregnancy and childbirth.

## Methods

### Study area

The study was conducted in Damot Gale district. Damot Gale is one of the thirteen district divisions of Wolayta Zone in SNNPR. The district has a total area size of 255.54 square kilometer, which is about 6.07% of the total areal size of Wolayita zone [16]. The district is located to the south central direction along the major road from Addis Ababa to Arbaminch. Bodit is an administrative town of the Damot Gale district. It is located 360 kilometers from Addis Ababa. Astronomically, the district is located between the coordinate of 6°32′24″N and 7°7′30″N latitude and 37°44′53″E and 37°56′24″ E of longitude. Mount Damot is the highest peak in the district with an altitude of 2800 meters above sea level within intermediate agro climatic zone of Woina Dega and Kola.

According to 2013 population projection of Ethiopia, Damot Gale district had a total population size of 186,687 (males 91,151 and females 95,536), of which, 142,971 were rural (males 69,908 and females 73,063) residents. The total number of children 6–59 months age residing in the rural areas of the district was 22,069. Malnutrition is one of the main health problems in South Regional State. It is predominantly seen among the rural population [4]. Thus, we selected the study area.

### Study design and study period

A community based cross-sectional study design was employed (February 1-March 2, 2016).

### Study participants

The study population was all children 6–59 months aged and their mothers in Damot Gale district. All children age 6–59 months old irrespective of ethnicity, religion and sex, were included in the study. Children 6–59 months of age and their mothers who did not live for at least 6 months in study area were excluded from the study.

### Sampling

The sample size was calculated by using simple proportion; with the assumption of prevalence underweight and wasting 28% and 7.6% respectively [4];95% of CI;5% of margin of error.

Since prevalence of wasting (7.6%) minimized the sample size, to increase sample size and minimize sampling error researchers took prevalence of underweight. Accordingly, the sample size was calculated as follows:

$$ \begin{array}{c}\mathrm{n}=\frac{{\left({\mathrm{Z}}_{\frac{\upalpha}{2}}\right)}^2}{{\mathrm{d}}^2}\Big[\mathrm{p}\left(1-\mathrm{p}\right)\\ {}\mathrm{n}=1.96\ *1.96*0.72*0.28/0.005*0.05\\ {}=309.8\end{array} $$

We considered design effect of 1.2 and 7% of non-response rate. The final sample size was 398 children aged 6–59 months. A two-stage cluster sampling design was used. In this sampling design, kebele was the primary sampling units and households (HH) with children 6–59 months along with their mothers were the secondary sampling units. Kebele is the administrative units under district. At first stage of sampling, Kebele were selected by using simple random sampling technique. The HH of the selected kebele were secured from the kebele health extension workers, and then searching for an eligible HH within each village of the kebele was done. The number of eligible HH was allocated based on population size using population proportional allocation technique. For HH with more than one eligible child, one child was selected by using lottery method.

### Data collection and measurement

The questionnaire was first prepared in English, translated into Wolayita and then re-translated back to English to check for its consistency. The questionnaire was developed referencing the 2011Ethiopian demographic health survey (EDHS). The questionnaire consisted of socio demographic and economic characteristics, environmental factors, child health and caring practice, behavioral factor, child anthropometry, and caring of mothers.

Eight data collectors and two supervisors were recruited. The data collectors and supervisors were trained for 2 days on the objectives of the study, definitions of terms and concepts, enumeration area reading, enumeration area delineation, selection of HH, handling of respondents, how to fill each part of the questionnaire, anthropometry measurement, and interview techniques.

After training, a pre-test was conducted on similar eligible study children from the neighboring enumeration area and applicability of procedures and tools were checked. Revision was done to the questionnaire depending on the pre-test result. The principal investigator and supervisors performed close site supervision during the whole period of data collection. Completed questionnaires were checked up daily before collecting from data collectors. The questionnaire contains the interview component and the anthropometric measurements.

### The interview component

Face to face interview was used for collecting detailed information about the eligible child and mother of the child from the child’s mother/care giver.

### The anthropometric component

In this component of the questionnaire, height and weight measurement of the child were taken. Digital weight scale was used to measure weight of children. Weight was recorded to the nearest 0.1 kg. Children who were unable to stand on the scale and below 24 months were weighed with the mother or caregiver, then the mother/caregiver was weighed alone, and the differences were used to obtain the net weight of the children. The height of the children was measured using a calibrated height measuring board. A child who cannot stand erected was measured in supine position (lying position). A child who can stand erected and above 24 month was measured standing against a calibrated height measuring board. The height measurement was taken to the nearest 0.1 cm.

### Variables

The variables selection was done based on the effect of the variables on malnutrition.

### Response variable

Wasting (weight for height) and underweight (weight for age) for children were used as the dependent variable. Children’s’ age, sex, height and weight were entered into WHO (world health organization) Anthro software to generate measurement indices of weight-for-age and weight-for-height. In this study, height and weight measurements of the children, taking age and sex into consideration, were converted into Z-scores. Thus, those below -2 standard deviations of the WHO median reference for weight-for age and weight-for-height were defined as underweight and wasted, respectively. The dichotomous variables underweight and wasting were defined as 1 = for underweight and 0 = for not underweight and 1 = for wasted and 0 = for not wasted, respectively.

### Explanatory variables

#### Socio-economic and demographic variables

Age of children, sex, family size, birth order, birth interval, maternal education,maternal occupation, marital status, agricultural land size, number of children 6–59 month in HH and main source for HH income.

#### Care of mother

ANC*,* using a skilled attendant at birth and family planning.

#### Child health and caring practices

IBF (initiation of breast feeding), exclusive breastfeeding, frequency of feeding, health status of the children, presence of diarrhea, fully immunized, eating with older sibling.

#### Environmental health condition

Occasions child’s mother washes her hands by using soap, availability of a latrine and sources of water supply.

### Data processing and analysis

After completion of data entry, recorded data of the WHO Anthro was exported to Statistical Packages for Social Science (SPSS) version 20 and recoded. Data obtained through survey questionnaire was also entered into computer for analysis using SPSS software. Accordingly, the data was edited, coded, and cleaned. Some consistency checks were verified by running frequencies and cross-tab. Description of frequencies, means, standard deviation and percentage were calculated by using SPSS. Bivariate analysis was performed to determine the differentials of underweight and wasting. Variables with *p*-value <0.2 in bivariate analysis were transferred to multivariate logistic regression. Odds ratio at 95% CI were used to measure strength of association between outcome and predictor variables. *P*-value < 0.05 was considered to declare statistical significance in multivariate logistic regression analysis.

## Results

### Socio-demographic factors

A total of 398 children aged 6–59 months were included, which makes the response rate 100%. Of the total 398 surveyed children, 134(33.7%) were found in the age group of 36–47 months. Among children covered in the survey, 43% were males and 57% were females. The large proportions, 365(91.7%) of the children’s mothers were married. Information collected on maternal educational status indicated that more than half of the children’s mothers (56%) were illiterate. The main source of HH income was agriculture for 85.4%. Children who were first births for their mother accounted, 56(14.1%) and 5+ birth orders accounted, 155(38.9%). Children born with a preceding birth interval below 24 month were 31.9%. A Large number (74.1%) of HH had one child. More than half of the HH (53.8%) agricultural land size was less than half a hectare (Table 1).

**Table 1:** Background characteristics of the respondents, Damot Gale, 2016

Characteristics(*n* = 398)	Categories	No.	%
Age	6–11 Months	36	9.0
	12–23 Months	113	28.4
	24–35 Months	79	19.8
	36–47 Months	134	33.7
	48–59 Months	36	9.0
Sex	Male	171	43.0
	Female	227	57.0
Maternal marital status	Married	365	91.7
	Separated	19	4.8
	Widowed	14	3.5
Maternal education	Illiterate	223	56.0
	Primary	129	32.4
	Secondary & above	46	11.6
Maternal occupation	Agriculture	314	79.0
	Off-farm	71	17.0
	Non-farm	16	4.0
Main source for HH income	Agriculture	340	85.4
	Off-farm	31	7.8
	Non-farm	27	6.8
Birth order	1	56	14.1
	2–3	73	18.3
	4–5	114	28.6
	5+	155	38 .9
Birth interval	First birth	56	14.1
	<24 month	127	31.9
	24–35 month	107	26.9
	36–47 month	61	15.3
	48 & above month	47	11.8
No of children 6–59 month in HH	1	295	74.1
	2–3	103	25.9
Family size	2–4	75	18.8
	5–8	284	71.4
	9–12	39	9.8
Agricultural land size	<0.5 hectare	214	53.8
	0.5–1 hectare	116	29.1
	>1–2 hectare	58	14.6
	>2 hectare	10	2.5
Occasions child’s mother usually wash her hand by using soap	before feeding the baby	90	21.9
	before breast feeding	37	9.0
	after cleaning baby’s bottom	160	38.9
	before food preparation	124	30.2
Type of latrine	Pit latrine	276	77.1
	Improved pit latrine	82	22.9
Main water source for drinking	River	31	7.8
	Public pipe	287	72.1
	Spring	80	20.1
Health status of children	Always sick	20	5.1
	Sometimes sick	108	27.1
	Healthy	270	67.8
Presence of diarrhoea	No	342	86.0
	Yes	56	14.0
Meal eating frequency per day	Twice per day	22	5.5
	Three time per day	201	50.5
	More than three times	175	44.0

About two in five (38.9%) mothers confirmed that they wash their hands with soap after cleaning baby’s bottom and one in five (21.9%) before feeding the baby. For 287(72.1%) HH, the main source of drinking water was public pipe. The majority of households (90%) had a latrine. A non- improved pit latrine was the most commonly utilized and it accounted, 276(77.1%). The health status of the children showed, 108(27%) sick some times. About 56(14%) of children had diarrhoea. Meal eating frequency per day showed that about half of children (50.5%) eat three times per a day (Table 1).

### Child health care and maternal care service utilization

One hundred sixth seven (42%) of children were not exclusively breastfed before 6 months of age. About 307(81%) of children were fully immunized. About 364(91.5%) of children did not eat with older sibling. About 141 (35.4%) of mothers did not attend ANC service during the index child pregnancy. Regarding place of delivery, only 109(27.4%) were delivered at health facility. More than half, 235(59%) mothers did not use family planning. Two hundred forty two (60.8%) of children fed the breast milk immediately after birth (Table 2).

**Table 2:** Child health care and maternal health service, Damot Gale, 2016

Characteristics	Categories	No.	%
Full immunization	No	72	19.0
	Yes	307	81.0
Exclusive breast feeding	No	167	42.0
	Yes	231	58.0
Eat with older sibling	No	364	91.5
	Yes	34	8.5
ANC	No	141	35.4
	Yes	257	64.6
Place of delivery	Health facility	109	27.4
	Home	289	72.6
Family planning	No	235	59.0
	Yes	163	41.0
IBF	Immediately after birth	242	60.8
	After 1 h	156	39.2

### Prevalence of malnutrition

Anthropometric measurements were done to determine the level of underweight and wasting. The study revealed that 27.6% and 9% of children were underweight and wasted respectively.

### Factors associated with malnutrition

#### Bivariate analysis

Factors associated with underweight on bivariate analysis included age of child, sex, agricultural land size, birth interval, eating frequency, health status of the child and ANC.

Maternal educational, maternal occupation, presence of diarrhea, age of the child, on ooccasions the child’s mother usually wash her hands by using soap, and ANC were associated with wasting.

### Multivariate analysis

#### Factors associated with underweight

According to multivariate analysis, age of child, sex of child, birth order, health status and ANC were associated with underweight.

The result revealed that sex of the child significantly affected the probability of underweight. Male children were 1.9 times more likely to be underweight than female children (AOR: 1.90; 95% CI : (1.10–3.32). Children with a shorter birth interval (below 24 month) were found 2.89 times more likely to be underweight than above 24 months birth intervals (AOR: 2.89: 95% CI : (1.23–6.80). The other predictor of underweight was health condition of children within past 2 weeks of study period. Children who had sickness some times for past 2 weeks were 59% less likely to be underweight than children who were sick in the past 2 weeks (AOR: 0.42; 95% (0.10–0.93). In this study attending ANC significantly reduced the probability of underweight. Children whose mothers attended ANC were 71% less likely to be underweight than children whose mothers did not attend ANC (AOR: 0.29; 95% CI: (0.16–0.52) (Table 3).

**Table 3:** Factors associated with underweight, Damot Gale, 2016

Characteristics	Categories	Underweight		COR at 95%	AOR 95% CI
		No (*N*, %)	Yes (*N*, %)		
sex	Female	181(62.8)	46(41.8)	1.00	1.00
	Male	107(37.2)	64(58.2)	2.35(1.75–3.15)*	1.9 (1.10–3.32)**
Birth interval (in month)	First birth	47(16.3)	9(8.2)	1.00	1.00
	<24	56(19.4)	71(64.5)	6.62 (3.93–11.13)	2.89(1.23–6.80)**
	24–35	88(30.6)	19(17.3)	1 .12 (.63–1.98)	0.77(0.31–1.89)
	36–47	56(19.4)	5(4.5)	0.46(0.21–0.99)*	0.38 (0.10–1.36)
	48 & more	41(14.3)	6(5.5)	0.76(0.36–1.58)	0.64(0.19–2.12)
Health status	Always sick	11(3.9)	9(8.1)	1.00	1.00
	Sometimes sick	80(27.7)	28(25.5)	0.42 (0.22–0 .81)*	0.30(0.10–0 .93)**
	Healthy	197(68.4)	73(66.4)	0.45 (0.24–0.82)*	0.37 (0.13–1.05)
ANC	No	69(24.0)	72(65.5)	1.00	1.00
	Yes	219(76.0)	38(34.5)	0.16 (0.12–0 .22)*	0.29 (0.16–0 .52)**

*p-value < 0.2, **p-value < 0.05

#### Factors associated with wasting

According to multivariate analysis, mother occupation, presence of diarrhea, and ANC were associated with wasting. Children whose mothers main occupation was non-farm were 7 times more likely wasted than children whose mothers main occupation were agriculture (AOR:7.06;95% CI: (1.31–38.21). Presence of diarrhea in the past 2 weeks prior to the survey was significantly associated with wasting. Children who had diarrhea in the past 2 weeks prior to the survey were 39.5 times more likely to be wasted than children who had no diarrhea (AOR:39.5,95% CI: (13.68–114.30). Children whose mothers attended ANC were 82% less likely to be wasted than children whose mothers did not attend ANC (AOR: 0.18; 95% CI :(0.07–0.45) (Table 4).

**Table 4:** Factors associated with wasting, Damot Gale, 2016

Characteristics	Categories	Wasting		COR at 95% CI	AOR 95% CI
		No (*N*, %)	Yes (*N*, %)		
Maternal occupation	Agriculture	285(78.7)	27(75)	1.00	1.00
	Off-farm	64(17.7)	6(16.7)	0.98(0.54–1.81)	1.06(0.41–2.74)
	Non-farm	13(3.6)	3(8.3)	2.43((1.03–5.75)*	7.06(1.31–38.21)**
Diarrhea	No	331(91.4)	11(30.6)	1.00	1.00
	Yes	31(8.6)	25(69.4)	24.26(14.39–40.91)*	39.55(13.68–114.3)**
ANC	No	120(33.1)	21(58.3)	1.00	1.00
	Yes	242(66.9)	15(41.7)	0.35(0.22–0 .55)*	0.18 (0.07–0.45)**

*p-value < 0.2, **p-value < 0.05

## Discussion

The current study showed the prevalence of underweight and wasting as 27.6% and 9.0%, respectively. This is relatively similar with EDHS study of SNNPR, which showed underweight as 28.3% and wasting as 7.6% [4]. But in current study, the prevalence of underweight and wasting were lower than the study of Hawassa Zuria, which showed underweight and wasting as 31.9% and 23.6%, respectively [13]. The prevalence of underweight and wasting in the current study is also lower than the study done in Mai-Aini Eritrean Refugees, which showed 33.4% and 24.6% of children were underweight and wasted respectively [17]. The difference may be due to the former study were conducted in refugee camp, as a result people are dependent on humanitarian aid.

Male children were more likely to be underweight than female children. This is similar with a study done in Bule Hora district of Oromia region and in the pastoral community of Dollo Ado district, Somali region [7, 8].

Children with shorter birth interval (below 24 month) were found more likely to be underweight than above 24 months birth intervals. This is consistent with other studies [9, 10]. The significant and higher risk of underweight among children of lower preceding birth interval might be due to close spacing of pregnancy. This finding suggested that family planning service should be strengthened at all levels for spacing the children.

Receipt of ANC was negatively associated with both underweight and wasting. This is consistent with other studies of developing countries [9, 11, 12]. It is also consistent with a study done in Nigeria, which showed children whose mother had fewer than four government ANC visits was more likely to have malnutrition [11]. This may be due to mothers who attend ANC may get valuable information about child care, health and nutrition. ANC visits are an indicator of contact with health services and health seeking behaviour, which may be associated with better care and feeding practices for young children. This is important for public health because it suggests another benefit of caring for women during pregnancy and child birth.

Another predictor of underweight was health condition of children within past 2 weeks of the study period. The children who had no sickness for past 2 weeks from study period were found to have decreased risk of underweight. This is in agreement with study conducted in Hawassa Zuria, which showed children who had no sickness for past 2 weeks from study period were found to have decreased risk of underweight than who were always sick [13]. This is probably due to decrease food intake (decrease appetite). It could be also due to a direct relationship of illness with malnutrition. Illness can lead to malnutrition and malnutrition predispose to illness.

In the current study, presence of diarrhea was significantly associated with wasting. This is consistent with the findings of another study [15]. It is also consistent with study done in Bule Hora disrtct of Oromia, which showed presence of diarrhea was associated wasting [7]. It is also documented that malnourished children have more diarrhea episodes and a child with diarrhea loses weight and can quickly become malnourished [10]. This could be due to s a reciprocal relationship of diarrhea with malnutrition, in which diarrhea predispose to malnutrition and malnutrition predisposing to diarrhea. This finding showed due concern should be given for diarrhea prevention at all levels.

Maternal occupation is another predictor, which affected nutritional status of children under five. Result of this study revealed that children whose mothers main occupation were non-farm were highly affected by wasting than children whose mothers’ main occupation were agriculture. This may be due to mothers whose occupation was farm may have the chance to get continuous food security than mothers whose occupation was non-farm. This finding implied that, support should be done in strengthening agriculture. In contrast to current study, study conducted in Oromia region of Ethiopia showed no significant effect on maternal occupation and wasting [18].

### Limitation of the study

As the study is cross-sectional in design, it neither represents seasonal variation of nutritional outcomes nor establishes causal relationship.

As the study was questionnaire-based, questions that required a good memory were vulnerable to recall bias.

The sample is only from rural areas, with particular environmental, political and personal conditions, and is thus difficult to generalize.

In this study some variables have wide confidence intervals; this may be due to low prevalence of the cases.

## Conclusion

The findings of this study indicate that malnutrition is still an important public health problem among children 6–59 months of age. High prevalence of malnutrition (underweight and wasting) was observed, indicating that the nutrition situation in study area is very critical.

Sex, birth order, health status of child and ANC were associated with underweight. Factors associated with wasting include mother occupation, diarrhea and ANC.

Availability and accessibility of ANC services to pregnant women should be increased as a means to improve long term nutritional and survival status of children.

Equal attention should be given for both males and females while feeding the Child.

Due attention should be given to initiate immediately breast feeding.

Prevention of diarrhea should be strictly done at community level.

Use of family planning should be encouraged at community level.

Health Extension workers should also strengthen giving participatory nutrition education to create awareness and to develop behavior change communication for better feeding and caring practices among the community. The six strategies that have been found to promote proper nutrition in a community should be implemented by all stakeholders: Basic education, healthy environment, maternal and child care, healthy social and family life, proper agriculture and public health measures.

## Abbreviations

ANC: Antenatal care
EDHS: Ethiopian demographic health survey
FMOH: Federal minster of health
HH: House hold
IBF: Initiation of breast feeding
SNNPR: South nation’s nationalities people region
WHO: World health organization
